# Preparation and Mechanism of Flame-Retardant Cotton Fabric with Phosphoramidate Siloxane Polymer through Multistep Coating

**DOI:** 10.3390/polym12071538

**Published:** 2020-07-12

**Authors:** Denghui Xu, Shijie Wang, Yimin Wang, Yun Liu, Chaohong Dong, Zhiming Jiang, Ping Zhu

**Affiliations:** Institute of Functional Textiles and Advanced Materials, College of Textile and Clothing, State Key Laboratory of Bio-Fibers and Eco-Textiles, Qingdao University, Qingdao 266071, China; 18561312665@163.com (D.X.); wsj960912@163.com (S.W.); 17863937942@163.com (Y.W.); liuyun0215@126.com (Y.L.); dongzhh11@163.com (C.D.)

**Keywords:** siloxane polymer, flame retardancy, protective char, cellulose, multistep coating

## Abstract

To improve the water solubility of phosphoramidate siloxane and decrease the amount of flame-retardant additives used in the functional coating for cotton fabrics, a water-soluble phosphoramidate siloxane polymer (PDTSP) was synthesized by sol-gel technology and flame-retardant cotton fabrics were prepared with a multistep coating process. A vertical flammability test, limited oxygen index (LOI), thermogravimetric analysis, and cone calorimetry were performed to investigate the thermal behavior and flame retardancy of PDTSP-coated fabrics. The coated cotton fabrics and their char residues after combustion were studied by attenuated total reflection infrared spectroscopy (FTIR-ATR), scanning electron microscopy (SEM), and X-ray photoelectron spectroscopy (XPS). All results presented that PDTSP-coated cotton fabrics had good flame retardancy and char-forming properties. PDTSP coating was demonstrated to posess gas-phase flame-retardant mechanism as well as a condensed phase flame-retardant mechanism, which can be confirmed by thermogravimetric analysis-Fourier transform infrared spectroscopy (TG-IR) and cone calorimetry test. Also, the preparation process had little effect on the tensile strength of cotton fabrics, although the air permeability and whiteness had a slight decrease. After different washing cycles, the coated samples still maintained good char-forming properties.

## 1. Introduction

Nowadays, functional materials, especially flame-retardant textiles, have attracted much attention along with an increase of public awareness of safety [1]. Worldwide, a large number of people die and lose fortunes in fire accidents due to the easy ignition of textiles every year [2]. Therefore, it is urgent to impart textiles with less flammability or nonflammability.

In the last decades, numerous researchers have focused on the investigation of flame-retardant textiles and various preparation methods have been reported [3]. Among these methods, sol-gel technique has been recognized as an excellent scientific approach to rendering textiles with different functional properties [4,5,6], such as flame retardancy [7,8], antibacterial properties [9,10], anti-wrinkle [11,12], and superhydrophobicity [13]. In this process, siloxane bonds are firstly hydrolyzed to silanol groups and then connected to cellulose substrate to form hydrogen and covalent linkages [14]. Recently, Alongi’s group has found that sol-gel derived coating can improve the flame retardancy of cotton fabrics by creating oxygen and heat transferring shield, hindering the formation of volatile species, and promoting the formation of protective char [15]. They have also demonstrated that effective flame retardant systems can be achieved by synergistic effects when sol-gel derived architectures are operating with phosphorus/nitrogen-containing functionalities. Diethylphosphatoethyltriethoxysilane (DPTES) and its combination with N-containing chemicals have been employed as flame retardants to form organic-inorganic hybrid coatings on the cotton fabrics [16]. The fire-safety properties can be significantly improved through increasing the char residue and hindering the production of volatile species, although these coatings can promote the thermal decomposition of cellulose.

To further investigate the synergistic effectiveness, some alkoxysilane monomers containing phosphorus and nitrogen elements have been synthesized and applied in the preparation of flame-retardant cellulose fabrics [17,18,19,20]. Xin and coworkers synthesized a novel Si/P/N-containing flame retardant and its flame retardancy on cotton fabrics was compared with Pyrovatex CP New [18]. The treated cotton fabrics showed excellent flame retardancy, which is similar to Pyrovatex CP New at the same concentration level except for formaldehyde release. Moreover, this flame retardant can be used as a semi-durable finishing agent for cellulose fabrics. Liu and coworkers prepared a novel Si/P/N-containing inorganic-organic hybrid coating (PPD-PTES) with sol-gel process and its flame-retardant mechanism was investigated [17]. Super flame retardancy can be obtained owing to the formation of a stable protective char layer and less release of flammable gases. This finding indicated that Si/P/N-containing FRs posess a gas-phase flame-retardant mechanism as well as condensed phase flame-retardant mechanism.

In our previous studies, some Si/P/N-containing chemicals were synthesized and used to prepare flame-retardant cellulose fabrics through the sol-gel method [21,22]. Although the flame retardancy can be greatly improved, a large amount of finishing additives and organic solvent used still restricted their application prospects. To avoid this problem, a novel water-soluble phosphoramidate siloxane polymer (PDTSP) was synthesized and deposited onto the cotton fabric by a multistep process to form organic-inorganic hybrid architectures with different coating layers. The flame-retardant properties and thermal stability were studied by a vertical flammability test (VFT), LOI, and thermogravimetric analysis (TG). Cone calorimetry and thermogravimetric analysis-Fourier transform infrared spectroscopy (TG-IR) were employed to investigate the flame-retardant mechanism of cotton fabrics with PDTSP coating. The structure of the char layer after combustion was inspected by attenuated total reflection infrared spectroscopy (FTIR-ATR), scanning electron microscope (SEM), and X-ray photoelectron spectroscopy (XPS). In addition, washing stability, tensile strength, air permeability, and the whiteness of PDTSP-coated cotton fabrics were measured.

## 2. Experimental

### 2.1. Reagents and Materials

Bleached cotton fabric was purchased from Qingdao Fenghuang Dyeing & Printing Co., Ltd. (Qingdao, China). Dimethyl phosphite (DTP), 3-aminopropyltriethoxysilane (APTES), and tetrahydrofuran were provided from Xiya Reagent (Chengdu, China). Carbon tetrachloride (CCl_4_) and triethylamine (TEA) were obtained from Sinopharm Chemical Reagent Co., Ltd. (Shanghai, China). All reagents were used without further purification.

### 2.2. Synthesis of Phosphorus/Nitrogen-Containing Siloxane Polymer (PDTSP)

The siloxane monomer (DTSP) was synthesized in our previous research [22]. Briefly, 0.5 mol of dimethyl phosphite and 0.5 mol of CCl_4_ were dissolved in 400 mL THF in a three-necked flask and the mixture was stirred for a while until the temperature cooled down to 0~5 °C in an ice bath. Then, 0.5 mol of APTES and 0.5 mol of TEA were added dropwise into the above solution. After complete addition, the solution was warmed up to room temperature and stirred for 8~12 h. The monomer DTSP was obtained after the removal of the hydrochloride salt of trimethylamine and solvent.

A polymeric form of DTSP was prepared as follows. In a 500 mL round flask, 0.5 mol of DTSP was dissolved in ethanol/water (1:2), and pH was adjusted to 4.0 with 0.1 M hydrochloride solution. After refluxing at 90 °C for 6 h, the siloxane polymer PDTSP was achieved by removing the solvent under reduced pressure. The structure of PDTSP was confirmed by NMR.

^1^H-NMR (400 MHz, DMSO) δ (ppm): 0.53 (2H), 1.48 (2H), 2.72 (2H), 3.37 (1H), 3.54 (6H), 5.02 (1H).

^13^C-NMR (100 MHz, DMSO) δ (ppm): 10.84, 25.50, 43.99, 52.71.

^31^P-NMR (DMSO) δ (ppm): 12.62.

### 2.3. Coating Procedure

Before coating, cotton fabrics were washed with tap water to remove some absorbed impurities and dry weight was measured after drying at 80 °C for 1 h. Then, 10% of PDTSP finishing solution in water with pH = 6.0 was prepared and cotton fabrics were immersed in the solution for 30 min with two dips and two nips. After padding with a pick-up of 100% under the pressure of 0.1 MPa, the samples were dried at 100 °C for 5 min and cured at 160 °C for 5 min. Finally, the dry weight of coated cotton fabrics was determined after washing with tap water and drying at 80 °C for 1 h. One and two layers of phosphoramidate siloxane polymer coating were deposited on the fabrics, which were coded as Cotton-PDTSP-1T and Cotton-PDTSP-2T. The add-ons of coated samples were calculated according to the previous study [21].

### 2.4. Measurements and Characterizations

^1^H nuclear magnetic resonance (^1^H-NMR), ^13^C nuclear magnetic resonance (^13^C-NMR), and ^31^P nuclear magnetic resonance (^31^P-NMR) were recorded on a Bruker AVANCE III HD 400 MHz spectrometer (Bruker, Germany) using DMSO as the solvent.

Fourier transform infrared (FT-IR) were collected on a Nicolet iS 50 FTIR spectrometer (Thermo Fisher Scientific, Waltham, MA, USA) using the ATR method in the range of 500~4000 cm^−1^ to analyze the coated cotton fabrics and char residue.

The surface morphology of PDTSP-coated cotton fabrics and their char residues was observed using a TESCAN VEGA3 scanning electron microscope (TESCAN, Brno, Czech Republic) with a magnification of 200 and 2000 (accelerating voltage: 10 kV).

X-ray photoelectron spectroscopy (XPS) spectra were recorded by an ESCALAB 250Xi instrument (Thermo Scientific, Walsham, MA, USA) equipped with Al Ka excitation radiation to measure the chemical composition of coated cotton fabrics and char residue.

A vertical flammability test (VFT) was performed according to GB/T 5455-2014 with a LFY-601A vertical combustion apparatus (Shandong Textile Science Research Institute, Jinan, China). The size of the tested fabrics is 300 × 89 mm^2^, and the ignition time is 12 s.

Thermogravimetric analysis coupled with Fourier transform infrared spectrometry (TG-IR) test was performed on the STA6000-Frontier (PerkinElmer, Walsham, MA, USA) analyzer at a heating rate of 10 °C/min with the temperature increasing to 800 °C under nitrogen atmosphere.

Cone calorimetry test of uncoated and DTSP-coated cotton fabrics was performed with a FTT0007 cone calorimeter (Fire Testing Technology, West Sussex, UK) according to ISO 5660 standard. For each sample, the fabrics are cut into 10 × 10 cm^2^, and three pieces are wrapped with aluminum foil and burned at an external heat flux of 35 kW/m^2^.

To measure the washing stability of PDTSP coated cotton fabrics, the samples were soaked in a conical beaker containing 0.15% detergent solution, and the beakers were fixed in a shaking water bath. The temperature was set at 49 °C and five washing cycles were defined as washing for 45 min with a rotation rate of 80 rpm. After 5, 10, 20, and 30 washing cycles, the samples were rinsed with distilled water, dried at 60 °C for 1 h. The flame retardancy of coated samples after washing was determined by VFT and LOI.

The whiteness of uncoated and Cotton-PDTSP-2T was tested by X-rite 8400 colorimeter (Grand Rapids, MI, USA). The air permeability of uncoated and PDTSP-coated cotton fabrics was measured by YG461E-III fully automata permeability instrument (Ningbo Textile Instrument Factory, Ningbo, China) with a pressure of 100 Pa, according to GB/T 5453-1997 method. Each sample was tested 10 times and the average value was obtained.

The tensile strength and breaking elongation of uncoated and coated cotton fabrics were measured according to GB/T 3923.1-2003 method. The size of the testing samples was 250 × 50 mm^2^. Each sample was tested 3 times and the average value was obtained.

## 3. Results and Discussion

### 3.1. Structural Analysis of PDTSP

**Figure 1:**
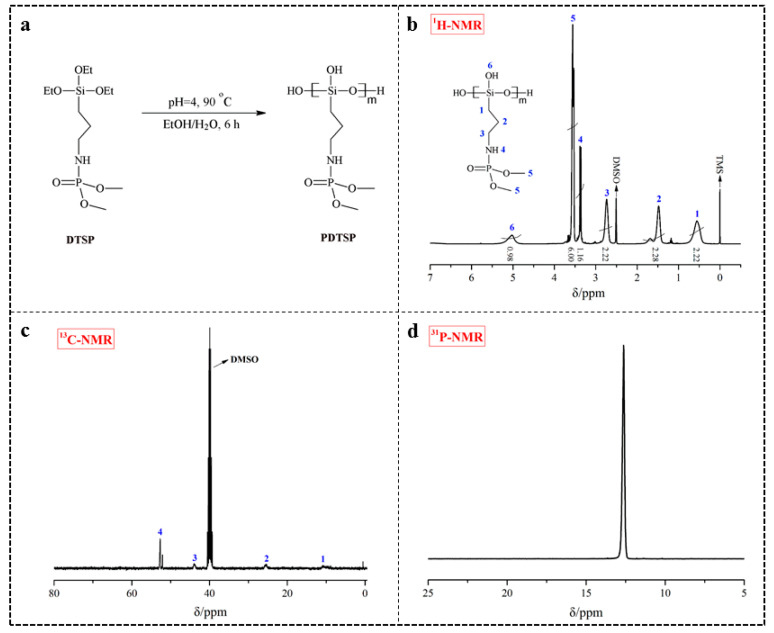
Synthesis route (**a**), ^1^H-NMR (**b**), ^13^C-NMR (**c**), and ^31^P-NMR (**d**) spectra of PDTSP.

The siloxane polymer PDTSP was prepared through the sol-gel method and the synthesis scheme was shown in Figure 1a. During this reaction, the siloxane bond (Si–OCH_2_CH_3_) in DTSP was firstly hydrolyzed to silanol bond (Si–OH), and then copolymerized themselves to form siloxane polymer, which can be reacted with the hydroxyl group (–OH) and bound onto cellulose. The chemical structure of PDTSP was confirmed by ^1^H-NMR, ^13^C-NMR, and ^31^P-NMR, presented in Figure 1.

As shown in Figure 1b for ^1^H-NMR, the multiplets at 0.56 ppm (labeled 1), 1.48 ppm (labeled 2), and 2.73 ppm (labeled 3) are attributed to the –CH_2_– groups (Si–CH_2_, C–CH_2_–C, C–CH_2_–N). The peak at around 3.37 ppm (labeled 4) corresponds to protons of the amino group. The –CH_3_ protons linked to phosphate appear as a doublet at about 3.54 ppm (labeled 5). In the PDTSP, the protons of siloxane bond (–Si(OCH_2_CH_3_)_3_) disappear and one multiplet appears at 5.03 ppm (labeled 6), which is assigned to the proton of Si–OH. Additionally, the integration ratio of 2.22:2.28:2.22:1.16:6.00:0.98 is given from these six groups under different environmental protons, which was close to the numbers of corresponding protons in PDTSP molecular (2:2:2:1:6:1). The peak at 2.50 ppm is attributed to the protons from the trace of DMSO. The ^13^C-NMR of PDTSP in Figure 1c has three multiplets at around 10.84 ppm (labeled 1), 25.43 ppm (labeled 2), and 43. 86 ppm (labeled 3), and one singlet at 52.71 ppm (labeled 4). A signal peak at 12.61 ppm in the ^31^P-NMR depicted in Figure 1d indicates that this compound has high purity. All these results indicate that PDTSP has been successfully synthesized.

### 3.2. Preparation and Characterization of PDTSP-Coated Cotton Fabrics

**Figure 2:**
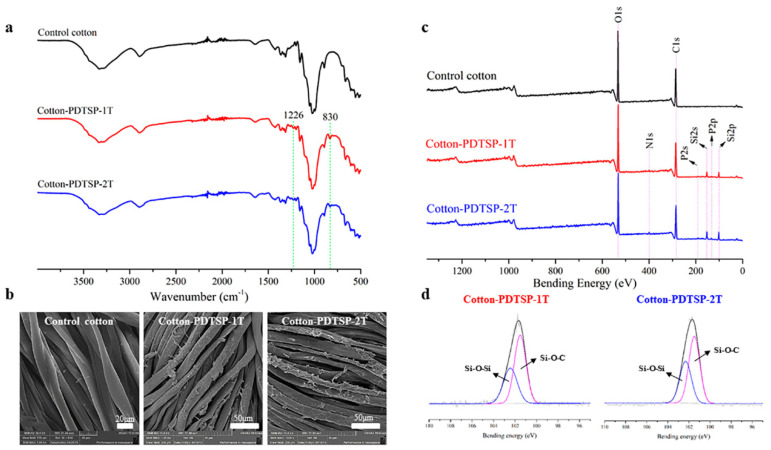
FT-IR spectra (**a**), SEM (**b**) and XPS (**c**,**d**) of Control cotton, Cotton-PDTSP-1T and Cotton-PDTSP-2T.

To confirm PDTSP successfully loaded on the cotton fabrics, the chemical state, elemental composition, and surface morphology of PDTSP-coated cotton fabrics were determined by FTIR-ATR, XPS, and SEM, which were presented in Figure 2. Compared with control samples, two new characteristic peaks in FTIR spectra (shown in Figure 2a) can be observed both in Cotton-PDTSP-1T and Cotton-PDTSP-2T. The absorption peaks at 1226 cm^−1^ and 830 cm^−1^ were attributed to the stretching vibration of the P=O and O–P–O group [23]. The surface morphology of control and PDTSP-coated cotton fabrics was measured by SEM and the photos were shown in Figure 2b. Compared with the smooth surface of control samples, there was an irregular film observed on the surface of PDTSP-coated cotton fiber. Additionally, the add-ons were summarized and the chemical state, such as silicon, phosphorus, and nitrogen, was also determined by XPS. The results were shown in Table 1 and Figure 2c. It can be seen that 8.8% and 13.8% of PDTSP can be loaded on the cotton fabrics for the sample of Cotton-PDTSP-1T and Cotton-PDTSP-2T, respectively. Three new peaks at 101 eV (Si2p), 191 eV (P2s), and 399 eV (N2s) in the XPS spectra of PDTSP-coated cotton samples could be observed, which was corresponding to the elements of Si, P, and N that existed on the surface of cotton fabrics. The high-resolution XPS spectra of Si2p for Cotton-PDTSP-1T and Cotton-PDTSP-2T were shown in Figure 2d, the binding energy range of 102–103 eV can be attributed to Si–O–Si, and the binding energy of Si–O–C was presented at 101–102 eV [24]. The presence of Si–O–C indicated a certain amount of covalent bond was formed between PDTSP and cellulose molecule. With the increase of treating times, the atomic content of carbon and oxygen decreased by the introduction of phosphorus/nitrogen-containing siloxane polymer. For Cotton-PDTSP-1T, the atomic % of Si, P, and N was 6.7, 1.14, and 1.25, which increased to 10.38, 1.24, and 2.26 in the sample of Cotton-PDTSP-2T. The co-existence of silicon, phosphorus, and nitrogen elements can render cotton fabrics with efficient intumescent flame-retardant property. All the above results indicated that PDTSP was successfully loaded on the surface of cotton fabrics.

### 3.3. Combustion Properties of PDTSP-Coated Cotton Fabrics

**Figure 3:**
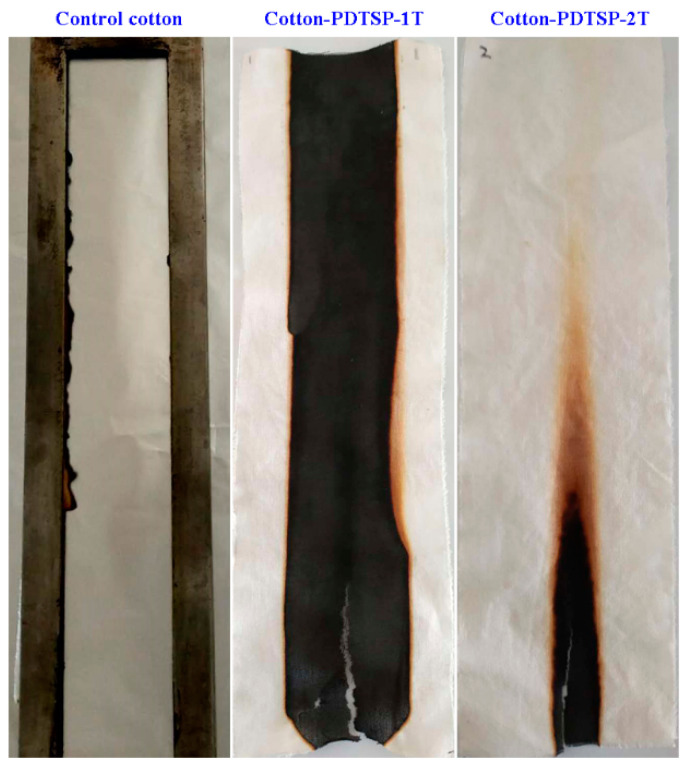
Digital photographs of cotton fabrics after VFT under different coating layers.

Vertical flammability test (VFT) and LOI was used to evaluate the combustion properties of cotton fabrics coated with PDTSP, and the photos taken after VFT and corresponding data summarized were given in Figure 3 and Table 2. It can be seen that control cotton samples burned completely, and no residue remained. After coating with PDTSP, the fabrics could leave considerable residue char and showed no afterglow time. Moreover, the afterflame time and char length decreased with the increase of treating times. Compared with the 5.1s of afterflame time for Cotton-PDTSP-1T, no afterflame time could be observed in the samples of Cotton-PDTSP-2T. Although Cotton-PDTSP-1T cannot pass the vertical flammability test and LOI was 24.1%, the char-forming properties were greatly improved. When the cotton fabrics were treated two times with PDTSP coating, the coated cotton samples presented a char length of 9.8 cm and LOI increased to 27.1%, which meant that Cotton-PDTSP-2T had good flame-retardant behavior and could pass the vertical flammability test.

### 3.4. Char Residue Study

**Figure 4:**
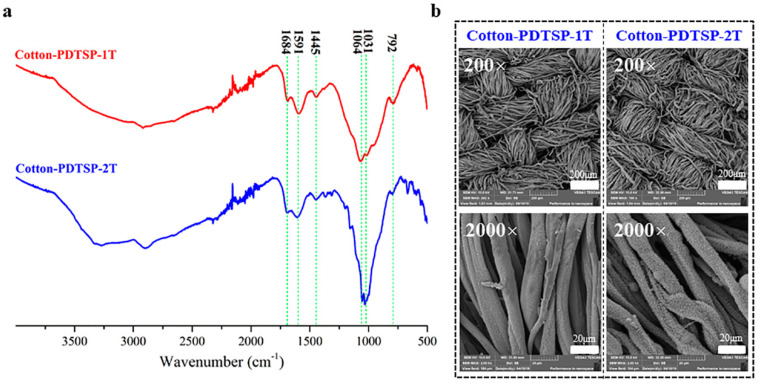
FT-IR spectra (**a**) and SEM (**b**) of char residues for PDTSP coated cotton fabrics.

To investigate the flame-retardant mechanism of PDTSP coating, the char residue of coated cotton fabrics after burning was measured by FT-TR, XPS, and SEM. As shown in Figure 4a, Both Cotton-PDTSP-1T and Cotton-PDTSP-2T samples showed characteristic peaks at 1684, 1591 and 1445 cm^−1^, which is assigned to the skeletal vibration of the aromatic ring and indicated aromatic carbonaceous structure formed during the combustion [25]. The peak at 1064 cm^−1^ was assigned to the stretching vibrations of P–O–P [26], which meant that polyphosphoric acid was formed during combustion. The formation of P–O–C can be proved by the characteristic peak at 1031 cm^−1^ [24]. The symmetric stretching of Si–O–Si can be observed at 792 cm^−1^ [26]. These results revealed that aromatic species and Si/P/N-containing architectures could be formed with the assistance of PDTSP.

SEM photos with different magnification of PDTSP-coated fabrics after VFT were given in Figure 4b. From low magnification (200×), it can be seen that the intact wave structure of fabrics could be reserved both in Cotton-PDTSP-1T and Cotton-PDTSP-2T. Under high magnification (2000×), lots of small-sized particles uniformly arranged on the surface of char residue can be observed, and more compact particles arrayed with the increase of coating times. These results indicated that PDTSP can form a protective layer on the cotton fibers, which can prevent the heat and oxygen entering into the underlying polymeric substrate from further combustion.

**Figure 5:**
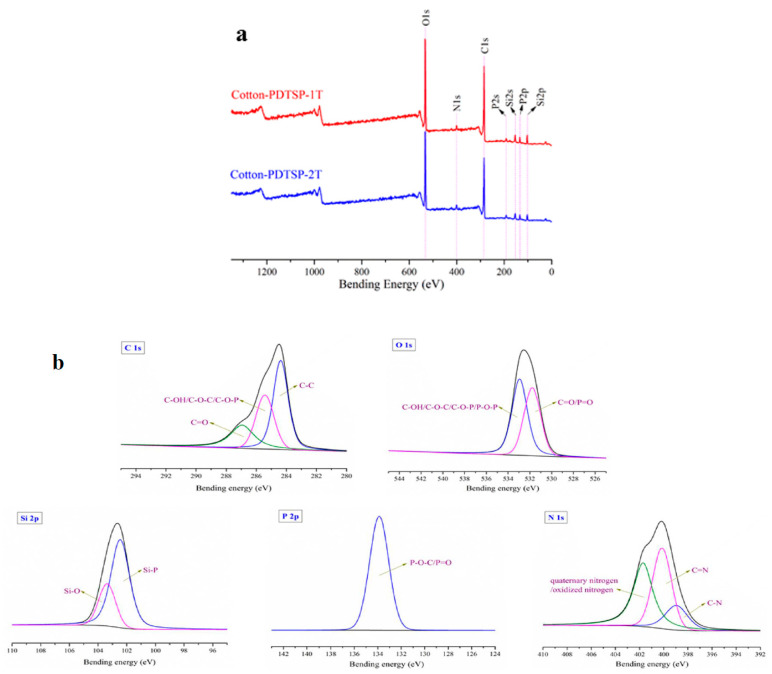
XPS spectra of char residue of PDTSP coated cotton (**a**); High-resolution C 1s, O 1s, Si 2p, P 2p, and N 1s spectra for the char residue of Cotton-PDTSP-2T (**b**).

The surface elemental composition of char residue was characterized by XPS and the results were given in Figure 5a and Table 3. The peaks at 103 eV (Si2p), 134 eV (P2p), and 400 eV (N2s) were corresponding to Si, P, and N elements. Compared with the unpyrolyzed coated fabrics (shown in Table 1), the contents of C, Si, P, and N increased after burning (shown in Table 3) by forming stable char and oxygen composition had a few decrease because of releasing volatile products and gases. The perfect retention of flame retardant elements in the carbon residue after combustion further indicates that the synergistic effect of silicon, phosphorus and nitrogen can provide high-efficiency intumescent flame retardant properties for cotton fabrics, which is consistent with the results of vertical combustion (show in Table 2). To further investigate the chemical composition of char residue, high-resolution spectra C1s, O1s, Si2p, P2p, and N1s for char residue of Cotton-PDTSP-2T was given in Figure 5. In the deconvoluted carbon C1s spectrum, three components were resolved. The binding energy at 284.4 eV assigned to the C–C group, while 285.4 eV was corresponding to C–OH/C–O–C/C–O–P, and binding energy at 286.9 eV was attributed to C=O groups [27]. The oxygen O1s narrow scan spectrum could be deconvoluted into two peaks, which were corresponding to C–OH/C–O–C/C–O–P/P–O–P at 532.9 eV and C=O/P=O at 531.7 eV [28]. These results were by FTIR spectra and indicated that polyphosphoric acid or phosphorous-containing char was formed, which could be supported by the P 2p spectrum (133.9 eV, P–O–C/P=O) [28,29]. Meanwhile, silicon also played an important role in the flame retardant and it can generate inorganic silicon dioxide and silicon-phosphorous compounds, which can be confirmed by the Si 2p high-resolution spectrum at 103.4 eV (Si–O) and 102.5 eV (Si–P) [8,27,29]. As a common gas-phase flame-retardant element, nitrogen still can form a stable structure in the char residue. In the N 1s high-resolution spectrum, the peak at 398.9 eV could be attributed to N–C, and 400.1 eV was due to existing of N=C structure [28,29,30]. Moreover, quaternary/oxidized nitrogen might be generated during combustion, which was proved by the peak at 401.7 eV [29]. In a word, PDTDP can promote the formation of a stable protective char layer and improve the fire-safety properties of cotton fabrics through isolating the heat and oxygen.

### 3.5. Thermal Stability

**Figure 6:**
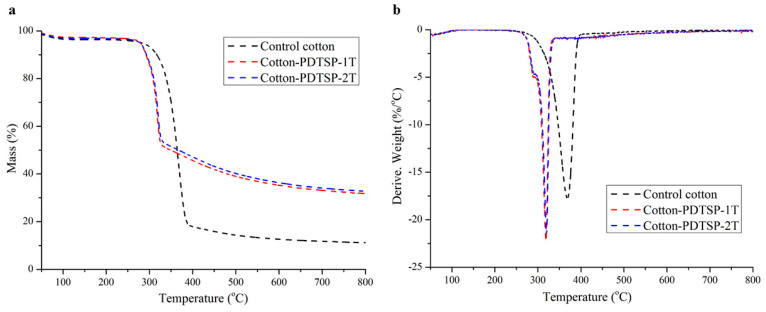
TG (**a**) and DTG (**b**) curves under N_2_ atmosphere.

The thermal stability of control cotton and PDTSP coated cotton fabrics was evaluated by TG and DTG in the N_2_ atmosphere, and the results were presented in Figure 6. As shown in Figure 6a,b, both Cotton-PDTSP-1T and Cotton-PDTSP-2T began to degrade at 279 °C (*T_5%_*, the temperature at 5 wt % weight loss) and the maximum rate degradation temperature (*T_max_*) appeared at 319 °C. These temperatures were lower than control fabrics and might be due to the dehydration of cellulose catalyzed by phosphorus/polyphosphoric acid generated from PDTSP during thermal degradation [31,32]. However, over 60% and 30% of char residue at *T_max_* and 800 °C could remain for Cotton-PDTSP-1T and Cotton-PDTSP-2T, and they were much higher than control cotton (43% and 11%), which indicated that PDTSP could greatly improve the thermal stability and char-forming properties of cotton fabrics.

### 3.6. TG-IR Analysis of Volatile Pyrolysis Products

**Figure 7:**
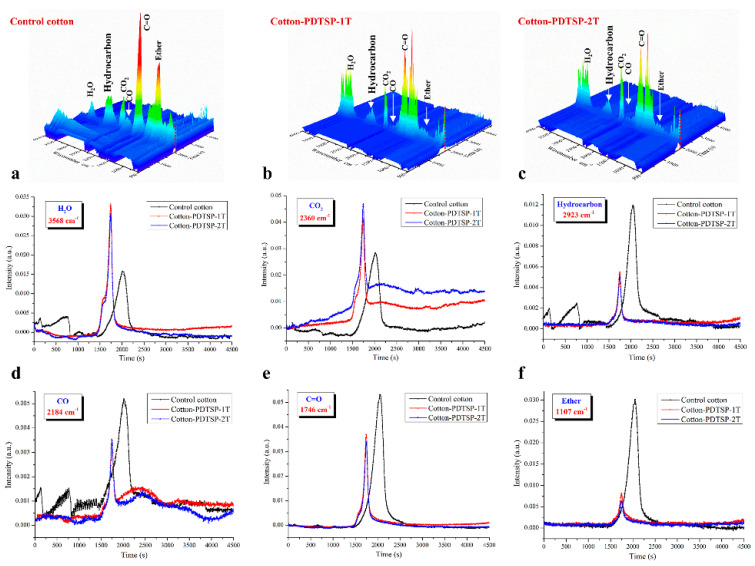
3D diagrams of gaseous volatiles, and intensity of typical pyrolytic products for Control cotton, Cotton-PDTSP-1T, and Cotton-PDTSP-2T.

TG-IR measurement was used to study the volatile pyrolysis products of control cotton and PDTSP-coated cotton fabrics during thermal decomposition. Cotton fabrics before and after coating decomposed to some characteristic products during heating, and 3D diagrams of gaseous volatiles during the pyrolysis process were given in Figure 7. The highest intensity of volatiles for Cotton-PDTSP-1T and Cotton-PDTSP-2T appeared earlier than control cotton, which might be related to the lower *T_max_* and by TG results. For control cotton, the typical pyrolysis products were H_2_O (3568 cm^−1^), CO_2_ (2360 cm^−1^), hydrocarbon (2923 cm^−1^), CO (2184 cm^−1^), C=O compounds (1746 cm^−1^) and ether (1107 cm^−1^) [21,33,34]. Moreover, the same products can be observed in the PDTSP-coated cotton fabrics, which means that PDTSP coating would not change the pyrolysis products and thermal-degradation process.

The absorption intensity of typical pyrolysis products (H_2_O, CO_2_, hydrocarbon, CO, carbonyl groups, and ether) corresponding to heating time was also investigated and the results were shown in Figure 7. Because of lower maximum rate degradation temperature, it took a shorter time for PDTSP-coated fabrics to reach the maximum absorbance intensities for all pyrolysis products. Compared with control cotton, lower intensities of flammable species such as ethers, CO, carbonyl compound, and hydrocarbon for PDTSP-coated cotton fabrics could be surveyed, which means that PDTSP coating could significantly decrease the release of flammable species feedback to the underlying material. Meanwhile, more nonflammable compounds like H_2_O and CO_2_ can be detected, these kinds of volatiles can enter into the gaseous phase and dilute the concentration of flammable gases. The above observation indicated that PDTSP coating owned a gas-phase flame-retardant mechanism to improve the flame retardancy of cotton fabrics.

### 3.7. Cone Calorimetry

Cone calorimetry is an efficient method to measure the combustion properties of materials in real fire circumstances. To further investigate the fire behavior and flame-retardant mechanism, the PDTSP coated cotton fabrics were detected by cone calorimetry, and corresponding data were summarized in Table 4. In this test, TTI (time to ignition), HRR (heat release rate), THR (total heat release), TSR (total smoke release), residue, and Av-EHC (average effective heat of combustion) were evaluated.

**Figure 8:**
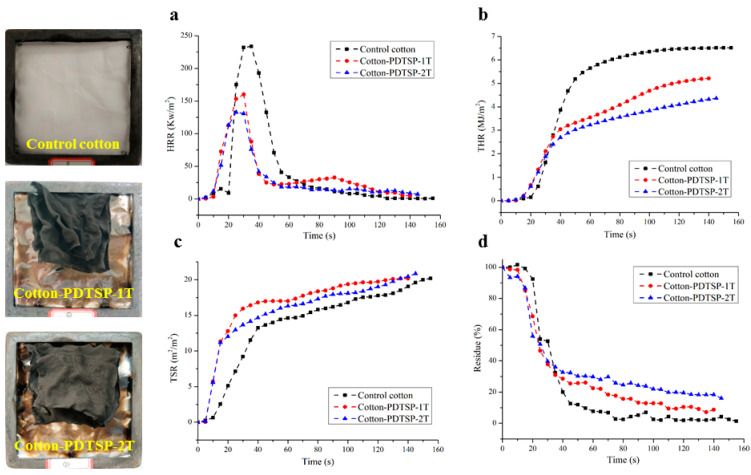
HRR (**a**), THR (**b**), Residue (**c**) and TSR (**d**) curves for Control cotton, Cotton-PDTSP-1T and Cotton-PDTSP-2T.

HRR and THR curves of samples were presented in Figure 8a,b. The values of PHRR (peak of the heat release rate) and THR were reduced after coating with PDTSP. Compared with control samples (PHRR, 233.80 kW/m^2^), Cotton-PDTSP-1T and Cotton-PDTSP-2T showed lower PHRR values at 160.09 and 132.89 kW/m^2^. Correspondingly, THR values decreased from 6.51 MJ/m^2^ to 5.21 and 4.36 MJ/m^2^. The reduction of PHRR and THR values indicated that PDTSP-coating can greatly hinder heat release and enhance the flame retardancy of cotton fabrics. It can be explained that PDTSP can promote the formation of the char layer and prevent further combustion by isolating heat and oxygen, which can be supported by char residue in Figure 8d. With the increase of PDTSP deposition, the char-forming properties were greatly improved. Compared with control samples (1.43%), higher residues were achieved for PDTSP-1T and Cotton-PDTSP-2T at 8.76% and 16.09%, respectively.

The smoke release was also investigated and TSR curves of control cotton and PDTSP-coated fabrics were depicted in Figure 8c. Compared with control samples, PDTSP coated cotton fabrics decomposed earlier and released more volatile products in the primary stages due to the catalysis of PDTSP, which can improve the flame-retardant properties by diluting the concentration of flammable gas and oxygen. However, the total smoke reduction (TSR) during the combustion had no significant difference before and after coating, and the average effective heat of combustion (Av-EHC) for cotton fabrics decreased from 15.54 to 11.21 and 9.81 MJ/kg for Cotton-PDTSP-1T and Cotton-PDTSP-2T, which indicated that PDTSP coating contained gas-phase flame-retardant mechanism by producing more combustible volatiles and less flammable volatiles [35].

### 3.8. Washing Stability

**Figure 9:**
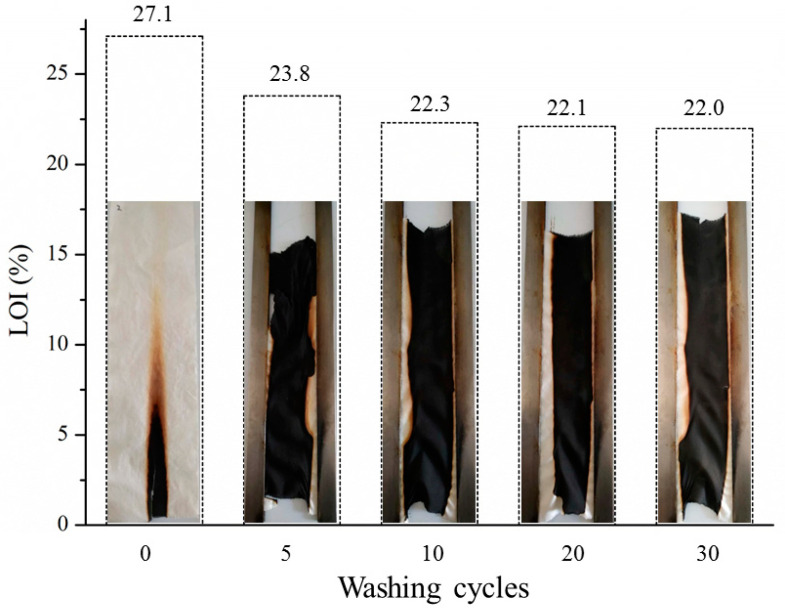
VFT pictures and LOI values of Cotton-PDTSP-2T against different washing cycles.

To measure the washing stability of Cotton-PDTSP-2T, VFT, and LOI values were detected and the results were presented in Figure 9. After five washing cycles, the LOI value decreased from 27.1% to 23.8%, which might be due to the removal of unbound PDTSP from the surface of cotton fibers and hydrolysis of Si–O–C bond between siloxane groups and cellulose [22]. With a further increase of washing cycles, the LOI values had a slight decrease and 22.0% of LOI value could be maintained after 30 washing cycles, which is higher than control cotton. From the VFT pictures, although the samples cannot pass the vertical flammability test after washing, they still had good char-forming properties and no afterglow phenomena could be observed during the test. To improve the washing durability, designing functional copolymers prepared by phosphoramidate monomer and siloxane monomer with multi-reactive groups might be an efficient way, which will be further studied in our following research.

### 3.9. Air Permeability, Whiteness, and Tensile Strength

The air permeability, whiteness, and tensile strength of control cotton and Cotton-PDTSP-2T were measured and the results were presented in Table 5. The air permeability of the PDTSP-coated cotton fabrics decreased by 25% compared with the control cotton, which indicated that PDTSP coating on the surface of cotton fabrics might reduce the air space between fibers. The whiteness of the control cotton was 97.2, which decreased by 44.1% after treating with PDTSP, which might be related to the color of PDTSP and higher curing temperatures.

The tensile strength and breaking elongation of cotton fabrics before and after coating were shown in Table 5. The results show that the tensile strength of Cotton-PDTSP-2T in the warp and weft direction had a slight decrease compared with the control cotton. However, the breaking elongation of PDTSP-coated cotton fabrics had a significant increase, which might be due to the stable cross-linking of PDTSP with cotton fabrics, giving the cotton fabrics good ductility [36]. The decrease of tensile strength might be related to the destruction of glycoside bonds of cellulose by the acid formed by hydrolysis of siloxane bonds and the high curing temperature [22].

## 4. Conclusions

A novel water-soluble phosphoramidate siloxane polymer (PDTSP) was successfully synthesized and characterized by NMR. The organic–inorganic hybrid architectures were deposited onto cotton fabrics with different coating layers through sol-gel technology. When two PDTSP layers were deposited, the coated cotton fabrics extinguished after the ignition source removed and passed the vertical flammability test. PDTSP coating can promote the thermal degradation of cellulose and greatly improve the formation of a stable char layer, which can protect cotton fabrics from oxygen/heat transfer and increase flame retardancy. Through TG-TR and cone calorimetry measurement, it can be indicated fewer less-flammable gases and less heat were released for PDTSP coated cotton fabrics. These results meant that this phosphoramidate siloxane polymer possessed a gas-phase flame-retardant mechanism as well as a condensed phase flame-retardant mechanism. Meanwhile, the air permeability, whiteness, and tensile strength of cotton samples were also investigated and the results showed that PDTSP coating had little effect on the physical and mechanical properties of cotton fabrics. Although the samples cannot pass the vertical flammability test after washing, good char-forming properties still can be maintained.

## Figures and Tables

**Table 1:** Add-ons and elemental composition of PDTSP-coated cotton fabrics.

Samples	Add-Ons (%)	Elemental Composition (%)				
		C	O	Si	P	N
Control cotton	0	64.37	35.63	-	-	-
Cotton-PDTSP-1T	8.8	56.77	34.14	6.7	1.14	1.25
Cotton-PDTSP-2T	13.8	55.72	30.39	10.38	1.24	2.26

**Table 2:** Results of VFT and LOI test of cotton fabrics with different layers.

Samples	Afterflame Time (s)	**Afterglow Time (s)**	Char Length (cm)	LOI (%)
Control cotton	28.5	30.4	-	18.4
Cotton-PDTSP-1T	5.1	0	≥30.0	24.2
Cotton-PDTSP-2T	0	0	9.8	27.1

**Table 3:** Elemental composition in the char residue.

Samples	Elemental Composition (%)				
	C	O	Si	P	N
Cotton-PDTSP-1T	59.94	28.68	6.33	3.53	1.52
Cotton-PDTSP-2T	58.14	29.44	6.47	3.25	2.7

**Table 4:** Cone calorimetry data for control cotton and PDTSP-coated cotton fabrics.

Samples	TTI (s)	PHRR (kW/m^2^)	THR (MJ/m^2^)	Residue (%)	TSR (m^2^/m^2^)	Av-EHC (MJ/kg)
Control cotton	17	233.80	6.51	1.43	20.19	15.54
Cotton-PDTSP-1T	9	160.09	5.21	8.76	20.18	11.21
Cotton-PDTSP-2T	10	132.89	4.36	16.09	20.87	9.81

**Table 5:** Tensile strength, air permeability, and whiteness of Control cotton and Cotton-PDTSP-2T.

Samples	Air Permeability (mm/s)	Whiteness Index (%)	Tensile Strength ~(N)		Breaking Elongation (%)	
			Wrap	Weft	Wrap	Weft
Cotton-control	407.2 ± 6.2	97.2	436.3 ± 12.3	357.5 ± 3.3	4.5 ± 0.1	25.8 ± 0.4
Cotton-PDTSP-2T	302.9 ± 2.7	54.3	386.5 ± 10.8	278.2 ± 3.5	7.2 ± 0.1	27.7 ± 0.4
